# A Systematic Review of Wheelchair and Mobility Scooter Containment Systems Used Internationally on Public Transit Buses

**DOI:** 10.3390/ijerph20206952

**Published:** 2023-10-20

**Authors:** Carolyn A. Unsworth, Amanda J. Timmer

**Affiliations:** 1 Institute of Health and Wellbeing, Federation University, Churchill Campus, Churchill, VIC 3842, Australia; a.timmer@federation.edu.au; 2 Department of Rehabilitation, Jönköping University, 55111 Jönköping, Sweden; 3 Department of Neurosciences, Monash University, Melbourne, VIC 3004, Australia; 4 Department of Occupational Therapy, James Cook University, Townsville City, QLD 4810, Australia; 5 Ramsay Health Care, Donvale Rehabilitation Hospital, Donvale, VIC 3111, Australia

**Keywords:** mobility device, disability, occupational therapy, WTORS, wheelchair tie-down and occupant restraint system

## Abstract

Despite the daily need for people to travel on public transit buses using their wheeled mobility devices, relatively little information is available regarding the most efficacious, affordable, and independent approaches to assist passengers with keeping their mobility devices in the designated wheelchair access space. A systematic review was undertaken to summarize this literature, place it within a geographical and temporal context, appraise its quality, and establish common themes. Key academic and grey literature transportation databases and government websites searched from 1990 to May 2022 identified 33 documents, which were appraised using the Mixed Methods Appraisal Tool (MMAT) or the Authority, Accuracy, Coverage, Objectivity, Date, Significance (AACODS) tool. Overall, the documents included were of good quality. The literature retrieved focused on the development and testing of the active containment systems favored for use in North America with a contrastingly small examination of the effectiveness of passive or semi-passive containment systems. Almost no literature was retrieved in English from European researchers documenting the use or effectiveness of rearward-facing passive systems. While tip or slide events are relatively rare among mobility device users, the effective use of containment systems is vital to minimize these. Further research is required to support transport policy makers, operators, and bus drivers to identify and correctly implement optimal containment systems to promote safety for all passengers on public buses.

## 1. Introduction

Public transit buses (over 22ft long), also variously referred to as transit buses or large accessible transit vehicles (LATVs), provide efficient and affordable transport with fixed route access into the community [1,2]. The proximity of boarding stops to people’s homes makes busing an ideal transport option for people with disabilities who use manual and powered wheelchairs and mobility scooters, collectively termed mobility devices throughout this paper. However, the safety of passengers using mobility devices as well as ambulant passengers during transit requires careful consideration. In most public buses, ambulant passengers are seated on benches or seating that is bolted to the floor and have access to handrails to hold onto, but do not use seat-belt style restraints. Passengers using mobility devices on public transit buses do not use seats secured to the floor, may not use a personal seatbelt to assist with remaining in their mobility device, and may not be able to hold onto rails or stanchion poles. This means the mobility device has the potential to slide outside of its designated position or tip over if sudden bus movements occur with sufficient force. Injuries to the mobility device user as well as other passengers may result from such events, although data on the frequency of slip or tip events and the number of injuries sustained by people using their mobility devices on public transit buses are relatively rare [3,4,5,6]. In the future, a rise in the number of adverse events may be seen as increasing numbers of older people seek to maintain their community access using mobility scooters [7,8]. This potential problem has been variously managed internationally [9] by using systems which can be classified as passive, or semi-active, or active tie-down systems which are also called restraint systems or referred to as wheelchair tie-down and occupant restraint systems (WTORS). In this paper, the term ‘containment’ system has been adopted as the collective term for any passive, semi-active, or active system.

Across Europe, passive containment systems are frequently in place, where mobility device users position themselves facing rearward, against a forward excursion barrier (FEB), often in the shape of an ironing board, and use a lateral stanchion pole or fixed or fold-down lateral excursion barrier (LEB) on the bus aisle side with a handrail on the bus wall side to reduce movement and offer support to hold onto [9]. This design appears to have been widely introduced following two German studies in 1992 by Glaeser and Kasten (summarized in English in Rutenberg and Hemily [9]), and a European Cooperation in Science and Technology (COST) study [10]. These studies determined that acceptable levels of safety were offered by the rear-facing position with a backrest and lateral aisle support (vertical stanchion) under normal bus operating conditions. More recently, a US study by Mather and Hunter-Zaworski [11] has also supported the effectiveness of this approach to passive containment of mobility devices, indicating that passive rear-facing systems are adequate for preventing tipping under ‘normal’ driving conditions, if the device has the back touching the backboard, the brakes applied, and the power off. In some current jurisdictions, a single mobility device tether belt is also offered, making this a semi-active containment system. For example, in some Australian buses, a tether belt extends from the bus wall at hip height, is looped by the person or companion around a part of the mobility device, and is then secured back into the buckle at the point of origin.

Active restraint systems, or WTORS, have been widely used in transit buses across North America [12] since the introduction of the Americans with Disabilities Act (1990). While it is mandated that WTORS be carried on public transit buses, their use is not. However, bus operators may require passengers of their service using mobility devices to use any WTORS installed [12,13]. Types of WTORS include three- or four-point tie-down strap systems, docking systems (where the wheelchair user has a pin under their seat and drives to position the pin in the docking box on the bus floor), manual clamps (the clamp attaches to one wheel), and automated or semi-automated systems where the user places themselves in the designated position, an automated lever locks against the wheels at floor level, and a sash and lap seatbelt is used [6].

Passive, semi-active, and active systems are associated with a range of advantages and limitations. Literature on the effectiveness of these systems to reduce or prevent sliding or tipping appears limited, and questions have been raised about their effectiveness in several studies, especially if the systems are not correctly applied [3,5,6]. However, it would appear that both systems can enhance passenger safety during transit. The different systems also offer different levels of user independence, with passive systems offering greater freedom. Active systems may include the use of strapping or bolts at floor level that could cause a trip hazard for ambulant passengers, and the costs associated with prospective or retrofitting active restraint systems can be high [14,15]. Finally, the need for a bus driver to assist in securing a person in their mobility device when using an active system may impact service delivery times and give rise to occupational health and safety concerns for bus drivers, as fitting these systems may place strain on the bus drivers’ knees and back [16,17]. Passengers may also be reluctant to have bus drivers lean over them while any personal seatbelts are applied. Therefore, a semi-active approach in which the user attaches the seatbelt if they can, or chooses to, may be preferable.

Internationally, there is interest in finding the optimal balance between user independence and passenger safety with respect to the use of containment systems on transit buses. For example, in Australia, neither system has been embraced, although many operators offer a rearward-facing travel option, and state jurisdictions are actively investigating the best option to use. Similarly in the USA, although WTORS have been in use for over 30 years, there has been considerable interest in the efficiency and effectiveness of passive containment systems [11,18,19]. In 2009, a summary from a workshop held in the United States in 2005 was published outlining the current status of science in wheelchair transportation safety [18]. This document also summarized Hobson’s 2001 review of international wheelchair safety standards, which is one of the few papers attempting to document the presence and use of standards on mobility device safety. However, both these documents are now over 10 years old, no quality appraisal has ever been undertaken of literature in this area, and a review documenting the nature and quality of this literature is timely. Therefore, the aim of this review was to systematically search and appraise the international literature on the use of mobility device containment systems on public transit buses. Specifically, the review aimed to summarize information retrieved, place this material within a geographical and temporal context, appraise the quality of the literature obtained, and establish common themes to describe and discuss this literature.

## 2. Method

This review used a systematic methodology and followed the Preferred Reporting Items for Systematic Reviews and Meta Analyses (PRISMA) guidelines [20]. A narrative approach was used in the analysis, as it was expected that the literature would be diverse in methodological approach, with a range of document types retrieved [21]. Both commercially published journal articles as well as reports retrieved from the grey literature were included in this review.

### 2.1. Search Strategy

Initially, a search for published literature was conducted using MEDLINE, CINHAL and Web of Science databases from 1990 to May 2022. The search commenced from 1990 to ensure the capture of all articles from the time the Americans with Disabilities Act (ADA) was introduced and the US research investigations at that time. The search terms used included both the MeSH and keywords of public transport* OR public transit bus* OR large accessible transit vehicle* OR LATV OR motor vehicle* AND wheelchair tie down* OR wheelchair tiedown* OR wheelchair tie-down* OR wheelchair occupant restraint system* OR wheelchair* OR wheelchair safety OR occupant restraint* OR wheelchair transport* safety OR WTORS OR wheelchair secure* OR passenger safety OR mobility aid restraint system* OR wheelchair secure* system*. The search strategy was developed for use in MEDLINE (Appendix A) and adapted for use in other databases. Next, a search of grey literature was undertaken that included a broad Google search, government websites, transportation-specific databases, and grey literature databases (Appendix B). The choice of databases for grey literature searching was directed by the published literature search results. Keywords were searched individually, and results were reviewed against the inclusion criteria.

### 2.2. Inclusion Criteria

Studies published in English in peer-reviewed journals after 1 January 1990, with any study design, were included in the white literature search. The grey literature search included government documents, technical and scientific reports, guidelines, and conference proceedings with full papers. Studies and other documents were included if the focus was on adults and the mobility device used was a powered or manual wheelchair or a scooter.

### 2.3. Excluded Studies

Documents were excluded if they were magazines or periodicals for consumer or trade groups (e.g., Exceptional Parent or OTPractice), standards or regulations, conference abstracts, annual meeting minutes, webpages, blog posts, or newspaper articles. We also excluded studies that did not demonstrate a 50% or more focus on public transit buses [4,22,23,24,25], a 50% or more focus on containment systems [3,26,27,28], or were an editorial or thesis document [29,30,31,32,33]. These excluded articles are listed to provide methodological transparency [34].

### 2.4. Article and Data Extraction

Articles from the database search were downloaded into Endnote and duplicates identified. Following detailed discussion on the inclusion and exclusion criteria, with 10% random examples drawn from the articles retrieved, the second author reviewed the search results and excluded articles based on title and abstract. Articles retained then had their full text reviewed against the inclusion criteria. The reference list of articles retained were also hand searched. A similar process was used for grey literature. All full-text articles identified through the database, hand searching, and grey literature search were then agreed upon for inclusion in this research by both authors. A Cohen’s unweighted kappa was performed to determine the inter-rater reliability of the full-text article screen [35], and disagreements were resolved through discussion. Using the guidelines from Hinkle et al. [36], a correlation coefficient of 0.90–1.0 was considered very high, 0.7–0.90 high, 0.50–0.70 moderate, and 0.30–0.50 low. Data were extracted from the included articles in the agreed categories of document author, date, place of publication, and methodology. In addition, agreed upon categories for mobility device containment systems described in the review were established.

### 2.5. Risk of Bias Assessment

Assessing the risk of bias of the included documents is an important part of a systematic review [37]. As a variety of document types were expected, two tools were used to assess the risk of bias. For documents where a research methodology was included, the Mixed Methods Appraisal Tool (MMAT) [38] was used. For documents with no or minimal research data, the AACODS (Authority, Accuracy, Coverage, Objectivity, Date, Significance) [39] was used. The MMAT enables bias ratings for studies from one of five types of research methods (qualitative, quantitative randomized controlled trial, non-randomized, descriptive, or mixed methods). Each scale has five different criteria which differ slightly for each research method. The AACODS tool includes several questions to evaluate the credibility of the document in each category. An AACODS category was considered a yes if the majority of questions within it were answered with yes. The two authors rated the documents independently. The reliability of the AACODS and MMAT scores of the two raters were calculated using an ICC (2,1) [40], and disagreements were resolved through discussion. Strength of reliability was again based on the guidelines from Hinkle, Wiersma, and Jurs [36].

### 2.6. Data Analysis

When planning this systematic review, it was anticipated that included studies would vary in document type, methodology, and quality. A narrative analysis was planned to examine how mobility device containment systems have developed over time and group articles together depending on their focus (e.g., design and development, review of application of standards, or stakeholders’ viewpoints). Documents were also analyzed according to their document type and quality using the risk of bias assessment. Arbitrary cut points were developed for both tools; for the MMAT, scores of 0–2 were described as low, 3 as moderate, and 4–5 as high, and for the AACODS, documents with two or fewer yes responses were considered to have low credibility, three or four yes responses were rated moderate, and high credibility was awarded to documents with five or six yes responses.

## 3. Results

### 3.1. Overview of Documents Retrieved

**Figure 1:**
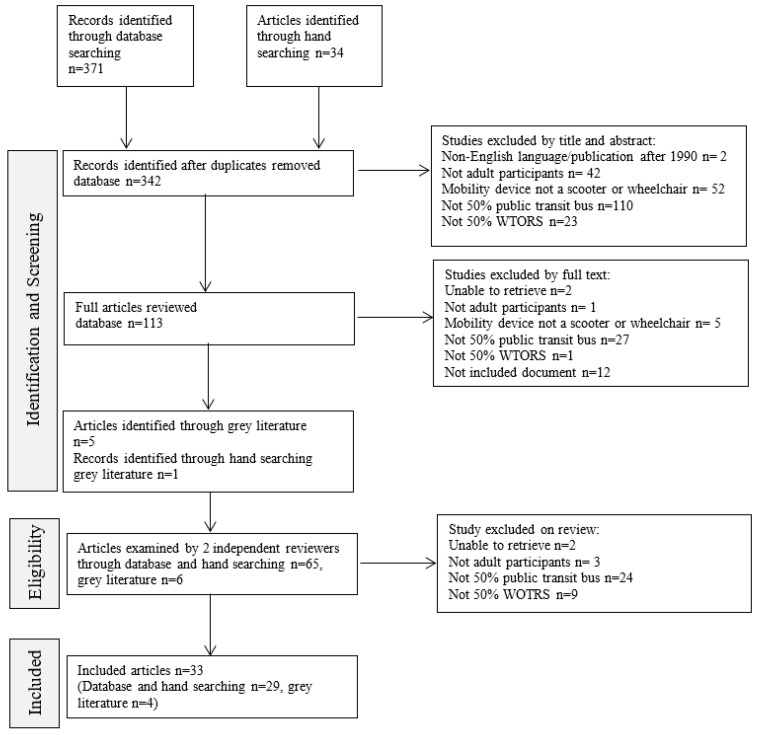
Flow diagram of study selection.

The database and hand searches were finalized in May 2022. The results of the search are presented in Figure 1 using a PRISMA format. Initial database searching resulted in 342 articles after duplicates were removed, including 34 articles sourced through hand searching and six from the grey literature search. After applying exclusion criteria, 33 documents were included in this review. Most documents were excluded because they did not have a 50% or more focus on public transit buses. After full text screening, an unweighted kappa between the two raters was found to be very high: Kappa = 0.875 (95% CI 0.769 − 0.981) · (78/83 = 94%) for inclusion of studies.

Table 1 presents the data extracted from the 33 included documents. Publication dates ranged from 1992 to 2022, with sporadic publications before 2006 and more consistent publications yearly from 2007. Peer-reviewed journal articles (n = 22) were retrieved more consistently in recent years, with reports from government or transport agencies (n = 11) published earlier. Only one document discussed scooters exclusively [41]. The type of containment systems described in the documents were categorized according to which way the passenger faces during the transport journey (front- or rear-facing), the system of securing the mobility device to the public transit bus (strap tie-downs, wheel clamps, aisle barriers, or docking systems) and any occupant restraint used (lap and/or shoulder seat belts). Appendix C provides color photographic examples of containment systems, noting that the photographs contained in the 33 retrieved documents were often of low quality that do not easily reproduce. No documents reported the use of a single tether belt/seatbelt for the mobility-device. It was found that front-facing systems were described more often than rear-facing ones (n = 27 vs. n = 16), and a greater number of documents discussed tie-down (n = 24) versus docking systems (n = 8). Where tie-down containment was described, shoulder/lap belts for occupant restraint were also noted (n = 25). Some documents described multiple systems (front-facing with tie-down containment; rear-facing and docking system) due to examination of current practices [15,18,42,43,44] or testing of different systems [2,14,19,45], or gathering opinions of people on the different systems [6].

The quality assessment of included documents is presented in the last column of Table 1. The reliability of the AACODS and MMAT scores between the two raters was found to be high: ICC (2,1) = 0.899 (95% CI 0.805 − 0.949), *p* < 0.001). The AACODS was used with 12 documents, all scoring high credibility, except for one document, which was rated as moderate [42]. On closer examination, most of the documents classified as grey literature were authored by research teams including experts in this field from universities or transport agencies, creating high-quality documents even without a peer-review process. The MMAT was used to appraise the remaining 21 documents, with the quantitative-descriptive methodological category used for 17 documents and mixed methods category used for four. The quality of these documents was more variable, with five rated as low-quality [16,46,47,48,49]. These studies lost points for their small sample sizes with limited representation and a lack of integration of qualitative and quantitative results in mixed-method studies. However, overall, the quality of the included documents was good, with 23 of the documents rated positively on either the AACODS or the MMAT.

All the documents were categorized into eight main focus areas, which have been summarized to explore the main findings: studies involving consumer opinion or observation, studies involving the testing of containment systems under normal driving conditions, studies involving bus drivers or transit operators, studies involving review of incidents, studies involving crash testing, reviews or discussions of standards, documents describing design and development of containment systems, and finally, literature based discussion of current practice. Initially, a discussion on how containment systems are used internationally is presented, together with an overview of documents describing the design and development of containment systems. Key findings in relation to these issues are summarized in Table 2 and further discussed below.

### 3.2. Design and Development of Containment Systems and Their Use Internationally

Most documents on system design included in this review originated from North America, with only four outside this geographical area from Brazil, France, and Germany [9,10,50]. The European Cooperation in Science and Technology (COST) Committee [10] reviewed the types of containment systems used in low-floor buses in Europe with the aim of informing transportation policy makers and providers in European countries of the benefits and risks associated with containment use. The report documented a preference for wheelchair users to travel unrestrained in designated spaces on buses. Evidence for the safety of this type of containment drew on earlier work from Germany in 1992 by Glaeser and Kasten (summarized in English in Rutenberg and Hemily [9]) which established forces during sharp accelerations of between 0.4 g and 1.0 g and tested the stability of wheeled mobility devices under these conditions with different containment scenarios. The COST Committee recommended that people with mobility devices travel rear-facing with the brakes on, the backrest in contact with an FEB, and holding onto an aisle support in the form of an LEB as the safest containment option. This type of containment was subsequently adopted across Europe, with almost no further research published. Additional work by Rutenberg [51] (authors unable to retrieve full document), Rutenberg and Hemily [9], and Rutenberg et al. [52] (authors unable to retrieve full document) in Canada supported the results of this European research and also led to the adoption of a rear-facing passive containment system in some Canadian transit systems at that time.

In the 1990s, most North American documents indicated support for, and use of, active containment systems. With the introduction of the Americans with Disabilities Act (ADA) in 1990, access to public transport for people using mobility devices, including buses, became mandatory [53]. The Department of Transportation, under the guidance of the United States Access Board, is responsible for enforcing the ADA through the Accessibility Guidelines for Transportation Vehicles, which were originally published in 1991 [54]. While both original documents provide guidance about the performance requirements for containment systems in buses (restraint forces required and limits of movement during normal conditions), the type of containment system to be used was not directed. Therefore, the development of containment options in North America was guided by universal use principles. This resulted in the four-point tie-down system, with shoulder/lap belts being viewed as optimal due to their low cost, ease of fit or retro-fit into buses, and the ability of this system to accommodate a wide variety of commonly used wheeled mobility devices and their occupants.

Several documents included in this review explore containment practices in North America with contrasting recommendations regarding the use of active restraint systems. As early as 1993 authors were providing an overview of the current use of active restraint systems and recommending changes to practice, including the need for universal containment, realistic standards development to guide the design of mobility devices and containment systems, and education on the use of such systems for the rehabilitation and transport communities [41,55]. Hunter-Zaworski and Zaworski [44] provided a critical review of containment issues following the introduction of the Americans with Disabilities Act, including the use of either belt-type containment or independent containment and the related standards to support the design and testing of these. The authors were critical of the time taken to develop standards for containment systems, which they noted had dampened the development of accessible travel on public transport. Recommendations were also made regarding the importance of including all stakeholders in the development of industry standards.

Nelson/Nygaard Consulting Associates (Easter Seals Project ACTION-Accessible Community Transportation In Our Nation) [48] subsequently reviewed practices at that time, and advocated for the continued use of forward-facing, four-point tie-down systems for containment but recommended more education and training for mobility device users and transport operators on their safe use as outlined in industry standards. The following year, Karg et al. [18] presented the outcomes from a stakeholder workshop aimed at reviewing and documenting the status of wheelchair transportation safety with a different view regarding mobility device containment. This report provided an overview of research on docking systems and rear-facing containment systems and suggested that these systems were the future of containment, but that limitations existed, including the docking system having issues with manufacturing and design, while rear-facing containment systems using an FEB and LEB (and with the possible addition of a mobility device tether attaching one point of the mobility device to the bus wall) lack an overarching industry standard to direct safety and design requirements. Finally, Hunter-Zaworski and Rutenberg [15] recognized the increasing use of larger and heavier mobility devices on LATVs and examined their safety during transit through a review of current practices internationally. Findings confirmed the use of four-point tie-down systems, in alignment with industry recommendations, is widespread throughout North America, whereas in other countries, a variety of rear-facing and docking containment systems can be found. In Europe, practice standards and guidelines are in use to support passive containment systems on transit buses, but aside from translations of the standards themselves, no reports on their use were identified in English for inclusion in this review.

Three of the documents reviewed specifically outline the design and development of containment systems; two focused on docking systems [56,57] and one on the development of an LEB to contain a mobility device from the aisle for rear-facing passengers [49]. All three provided information about the design requirements of the system, including engineering, performance, and stakeholder views, including transit operators and manufacturers. Both Hunter-Zaworski et al. [57] and Hobson and van Roosmalen [56] also gathered information from mobility device users as experts on the emerging designs.

### 3.3. Consumer Opinion or Observation of Consumers Using Containment Systems

Consumers were included in 15 of the 33 documents. In five documents, consumer opinion was included as only a small part of the report and not considered in this summary [15,41,47,56,57]. Five documents surveyed consumers regarding their opinion on different aspects of mobility devices on buses. Unsworth et al. [6] surveyed 448 mobility device users and ambulant passengers in both the US, where active containment systems are widely in use, and in Australia, where the most common containment option is to travel rearward-facing with an FEB. These authors aimed to gather information to support the introduction of a more uniform approach to containment systems in Australia and reported that 92% of respondents thought a containment system should be used but that active systems were only rated 7.66/10 as effective to prevent injuries. Only a minority of respondents using mobility devices (13.2%) had ever slid or fallen while in transit, or seen a person slide or fall (13.6%, for ambulant passengers), with no differences between countries despite the rarity of active containment systems in Australia. Brinkey et al. [58] administered a survey to a convenience sample of 107 wheelchair users to establish their knowledge about safe wheelchair transport practices. The majority of respondents (63%) reported using the tie-down containment method but had received little or no education about the proper use of containment or restraint systems in public transport, and less than 1% of wheelchair users had knowledge that industry standards existed to guide best practice. A further three documents surveyed consumers about active securement systems and their experiences of using these on buses [13,48,50]. In all three documents, consumers reported lack of use or misuse of containment systems, with two also reporting that the most common reason for this related to lack of time or knowledge of the bus driver [13,48].

Wolf et al. [59] observed 26 cases of active containments system usage on LATVs over a six-month period, demonstrating that tie-down containment was used appropriately in only 10 (38%) instances. At other times, fewer than the available four tie-downs were used, or no occupant restraint system was employed, increasing the risk of injury to mobility device users or other passengers. Similarly, Frost Bertocci and Salipur [12] and Salipur, Frost, and Bertocci [60] reported on the same dataset of retrospective reviews of on-board video footage of 295 video recordings and noted that 73.6% of mobility devices were unsecured during transit. These studies are reviewed later as they also report incident data. Finally, two documents asked consumers to test different types of containment and comment on ease of use, comfort, security and independence [2,45]. Consumers in the study by Perez et al. [2] trialed three- and four-point forward-facing containment systems and an automated rear-facing active containment system and indicated that the automated rear-facing containment system was more time-efficient, easier to use, and allowed for increased independence. However, users did have reservations about travelling rearward-facing, with difficulties seeing approaching bus stops and concerns about motion sickness and discomfort. Consumers in the study by van Roosmalen et al. [45] indicated that a forward-facing prototype autodocking system they tested (pneumatically operated attachment at the rear of the mobility device) was preferable to a four-point tie-down system or the rearward-facing prototype autodocking system (pneumatically operated elbow locking the mobility device in place at wheel level), as it was not only quick and easy to use, but allowed for forward-facing travel.

### 3.4. Testing of Containment Systems under Normal Driving Conditions

Several documents considered the performance of containment systems during normal driving conditions. Both Hobson and van Roosmalen [56] and Turkovich et al. [19] tested different types of containment (four-point tie-down, autodocking, and rear-facing containment with FEBs and LEBs) for displacement of the wheelchair during turning and breaking conditions. All devices in all driving situations moved less than the ADA-recommended movement of 51 mm. In addition, Turkovich et al. [19] reported that accelerations during normal LATV driving, hard braking, and rapid turning did not exceed 0.76 g, again less than the ADA-recommended maximum of 1 g of force, noting that these recommendations are now over 12 years old, and technology has enabled much smoother driving with lower forces generated. However, at that time, the authors recommended that systems other than the ADA-recommended tie-down system may also be able to provide safe containment of mobility devices under normal driving conditions. Three documents specifically examined the performance of rear-facing containment to limit movement out of the wheelchair access space. Zaworski [49] conducted field testing of a prototype LEB on an LATV with a manual wheelchair and a scooter in a rear-facing position with an FEB. The testing produced little or no side movement from either the scooter or the wheelchair, with the author recommending the use of the LEB with rear-facing containment to prevent wheelchair tipping and rotation into the aisle. Hunter-Zaworski and Zaworski [47] and Mather and Hunter [11] tested rear-facing containment in buses for movement during normal driving conditions, and their results also supported the use of rear-facing containment for wheeled passengers, providing that the device is backed up to an FEB (backrest in contact with the FEB), the brakes are applied, there is use of aisle side containment (LEB), and the device fits inside the containment area. However, Mather and Hunter-Zaworski [11] also noted that the driving styles of the two bus drivers in their study produced different mobility device movements during on-road testing, with the more experienced driver producing smoother turns and less mobility device movement, concluding that driver style may impact safety. Finally, Wolf and colleagues [59] used computer simulations to test three different containment configurations (four-point tie-down with no seatbelt, two-point tie-down with no seatbelt, and two-point tie-down with seatbelt) with a manual wheelchair during breaking and turning emergency conditions. These containment configurations were intended to simulate common misuse scenarios as evidenced in earlier research (e.g., Buning et al. [13]). Results indicate that full or partial tie-down, without an occupant seatbelt, could result in unsafe conditions, even during normal driving. It was noted that the addition of an occupant seatbelt with partial tie-down could increase safety under breaking but not during turning conditions.

### 3.5. Bus Drivers’ or Transit Operators’ Perspectives on Containment Systems

Five documents considered containment system use from the perspective of bus drivers or transit operators. Two teams [9,14] considered rear-facing containment (including FEB and LEB), with operators responding positively to using this type of containment. Ahmed et al. [16,46], in two related studies, conducted an ergonomic analysis of bus drivers using a four-point tie-down system to identify possible risks for drivers. Findings indicate a significant mismatch between securement requirements, and bus drivers’ functional limitations, which may create an unsafe environment. Scooter use on buses was examined by Spiller [41], who surveyed 28 transit operators regarding operational issues in containing or restraining three-wheeled scooters. Bus operators reported multiple problems, including lack of training, risk of injury to drivers, variable types of scooters with limited securement points, and the issue of passengers refusing to have their mobility device secured. Operators suggest that mandatory standards need to be developed that guide the design and manufacturing of scooters, training, and best practice application of the existing three- and four-point tiedown system of containment and the exploration of other containment options for scooters. Seven years later, Hardin et al. [17] also called for further direction concerning the requirements for containment through mandatory and voluntary standards in the United States. These authors conducted a comprehensive review of containment issues for transit operators by distributing a survey to 270 transit agencies, with 95 (35%) responses, asking for information in the following areas: general agency information, containment equipment, mobility device accommodation challenges and strategies, containment-related complaints, operator training, and maintenance of containment equipment. The two most common containment systems installed on transit vehicles in this 2002 survey were the wheel-lock device (42%) and three- or four-point tie-down with seat belt systems (94%), with the tie-down system being the most used by passengers. The respondents reported on the advantages and disadvantages of the different containment systems and noted that injuries to both passengers and bus drivers when using or not using active four-point tie-down restraint systems were identified. Overall, the authors found that issues with containment are prevalent and increasing in scope mostly due to the increased use of mobility devices with unique designs that make the identification of points for securement difficult, or even impossible. They reported that the challenge for transit operators is “to locate and install containment equipment that is ADA-compliant, can accommodate a wide and ever-growing variety of mobility devices, assures at least a sufficient measure of safety to all passengers, and will not harm the [mobility devices] used by passengers” (p. 46).

### 3.6. Review of Incidents Involving Mobility Device Users

Four documents reviewed incidents relating to people using mobility devices when in transit on buses, using two methodological approaches. Spiller [41] and Shaw and Gillispie [5] conducted literature reviews of incident data and used interpretations and estimates to identify mobility device user risk aboard large transit buses during crash situations. Neither author could find any substantial literature on crash incidents and outcomes, suggesting that this research is not a priority given that large transit vehicles are involved in relatively few crashes and mobility device users form a small proportion of all passengers. More information was located concerning incidents that occurred under normal driving conditions, and the authors suggested that the level of impact protection recommended by the ADA may be too high (able to withstand forces of 8–10 g), that alternative containment systems should be explored, and that further research in this area is required [5].

Salipur et al. [60] and Frost et al. [12] reported on reviews of video footage from the same dataset of 295 wheeled mobility device trips over a 21-month period with forward-facing four-point containment systems with shoulder/lap belts, which were conducted to identify containment configurations associated with adverse incidents. The authors found that adverse events did occur during normal driving conditions and that misuse (tie-down correctly used but misuse of the seatbelt system, n = 22, 7.5%) or no containment (n = 273, 92.5%) was common. The most common misuse was the use of the lap belt strapped around the back of the wheelchair or scooter to secure the mobility device. Scooters were found to be the mobility device least likely to be properly secured. The authors recommended the development of containment systems that increase stability and can be independently used, together with increased training for bus operators in the correct use of active containment systems on LATVs.

### 3.7. Crash Testing of Mobility Device Containment Systems

Hunter-Zaworski and Zaworski [47] reported research regarding front impact crash tests between 40 ft-high floor buses and three different car types, all weighing the same, and the impact on mobility device movement (sliding or tipping). The cars were towed into the front of the buses at a speed of 30 mph. The aim was to compare actual acceleration rates with the recommended 20 g acceleration that is used in the ANSI/RESNA wheelchair containment standard WC-19. Results indicate that the actual acceleration rates observed are substantially lower than the standards recommend for crash testing, impacting the testing of containment options. The authors concluded that if the occupant cannot hold onto a rail, manual wheelchairs and scooters are likely to move, and that rear-facing containment with an FEB but no LEB is likely to cause a wheelchair to tip over under extreme conditions. The use of sled testing was reported in two documents to test the stability of mobility devices when using a docking device [56] and during rear impact collisions [61]. Hobson and van Roosmalen [56] completed research with 48 kph, 20 g frontal-impact sled testing and a three-point tie-down to measure the amount of movement the containment device allowed. Minimal movement was observed, and the system was recommended for commercial use. Salipur and Bertocci [61] used rear-impact sled testing with three manual wheelchairs (test dummy restrained by a three-point lap and shoulder belt and the wheelchair secured by a four-point tie-down system) to compare the forces sustained by containment systems during frontal and rear impacts. The rear-impact sled testing produced substantially higher loads, particularly in the lap belt, suggesting this is an important consideration when designing containment systems to protect wheelchair users during rear-impact collisions.

### 3.8. Review or Discussion of Standards and Their Application

The development of practice standards to guide the design and testing of containment systems in the United States did not occur until some years after the introduction of the ADA and were voluntary. While it is not within the scope of this systematic review to examine the development and use of these practice standards, five documents included in this review provide commentary or examination of the voluntary standards relevant to active containment systems in the United States [42,43,62,63]. A review of these documents in chronological order assists in understanding the impact that the introduction of standards had on the development of containment systems in the United States. While Schneider et al. [63] and Buning et al. [62] provide commentary on the use of voluntary standards, Frost et al. [43] provides a more critical review of the limitations of the standards, in particular the overly conservative design and testing requirements for containment systems in LATVs. These authors recommend the development of new standards, specifically for low-force LATV environments; best practice guidelines to help educate passengers and vehicle operators concerning the correct use of securement systems and how to use a mobility device on public transport; training tools and random monitoring of transit operators regarding appropriate use of containment systems and implications of misuse; disability awareness training for vehicle operators; a certification process that identifies transit operators with appropriate training; and modifications to the ADA to take into account the different vehicle environments being used by mobility devise users. Subsequently, Cross [42] provided an overview of the major changes to the revised ADA and accessibility guidelines and proposed changes that were not adopted. Regarding containment systems, the changes included reducing the minimum design load of containment systems in large transit vehicles and allowing the use of rear-facing systems with aisle barriers in these vehicles. Proposed side-facing containment was not adopted.

**Table 1:** Summary of included articles.

Author Name, Date, and Country of Origin (Arranged by Publication Date from Oldest to Most Recent)	Aim or Purpose, Document Type	Methodology	Containment System Described (Forward- or Rear-Facing, Tie-Down Straps, Wheel Clamps, Docking System, FEB, Aisle Barrier in the Form of an LEB, Automated LEB, Seatbelts Including Lap Belt with/without Shoulder Belt, and Mobility Device Tether).	Key Focus Area *	Risk of Bias (Highest Score Possible) AACODS (6)/MMAT (5)
Hunter-Zaworski et al. (1992) North America [57]	To design and test a wheeled mobility device containment docking system, Report	Design and development, in-vehicle testing	Forward-facing, docking system, lap belt	7, 8	AACODS 6/6
Hunter-Zaworski and Ullman (1993) North America [55]	To explain the mechanics of containment and restraint systems for mobility devices, Peer-reviewed journal	Review of current containment and restraint technology	Forward-facing, 2-, 3-, 4-point strap tie-down, wheel clamps with wheel straps, lap belt with/without shoulder belt	6	AACODS 6/6
European Cooperation in Science and Technology (COST) Committee (1995) France/Continental Europe [10]	To gather information on current European operational experience of low-floor buses and provide guidance on best practices, Report	Review of current research	Rear-facing with FEB, LEB	6	AACODS 6/6
Spiller (1995) North America [41]	To provide an assessment of containment and restraint issues related to the transport of tri-wheeled scooters and their occupants, Report	Review of current research and standards, survey of stakeholders	Forward-facing, 2-, 3-, or 4-point tie-down straps, rear wheel clamp	2, 4, 5, 6, 7	AACODS 6/6
Hunter-Zaworski and Zaworski (2001) North America [44]	To review the current status of wheelchair containment in public transit vehicles, Report	Review of current standard development	Forward-facing, 4-point strap tie-down, lap and shoulder belt Rear-facing, docking system	6	AACODS 6/6
Hardin et al. (2002) North America [17]	To establish the containment issues currently facing transit agencies, Report	Quantitative-descriptive (survey)	Forward-facing, 4-point strap tie-down	4	MMAT 4/5
Rutenberg and Hemily (2003) North America [9]	To review the state of practice with rear-facing containment systems across USA, Canada, and the world, Report	Review of current literature and standards worldwide and quantitative-descriptive (interview and survey)	Rear-facing with FEB, LEB, mobility device tether	4, 6	AACODS 6/6
Shaw and Gillispie (2003) North America [5]	To identify wheelchair rider risk on large transit buses and characterize the transit bus crash environment in terms of severity, principal impact direction, and frequency of occurrence, Peer-reviewed journal	Quantitative-descriptive (Literature review of accident data)	Forward-facing, 4-point tie-down, lap and shoulder belt	2	MMAT 5/5
Hunter-Zaworski and Zaworski (2005) North America [47]	To evaluate rear-facing wheelchair containment and investigate wheelchair and wheelchair-user response to this method, Report	Quantitative-response testing, descriptive (survey)	Rear-facing with FEB, LEB	1, 3, 7	MMAT 2/5
Buning et al. (2007) North America [13]	To identify the factors that contribute to low use of active containment systems by wheelchair users, Peer-reviewed journal	Quantitative-descriptive (survey)	Forward-facing, 4-point tie-down, lap and shoulder belt	7	MMAT 5/5
Hobson and van Roosmalen (2007) North America [56]	To develop and test an auto docking device, Peer-reviewed journal	Quantitative-design and development, performance testing, and user evaluation (focus groups)	Forward-facing, docking system, lap belt	1, 3, 7, 8	MMAT 4/5
Wolf et al. (2007) North America [59]	To evaluate active containment system usage and to evaluate wheelchair and occupant response under emergency driving conditions when using less than optimal containment, Peer-reviewed journal	Mixed Methods-Qualitative (ethnographic) and Quantitative (computer simulation)	Forward-facing, 4-point tie-down, lap and shoulder belt	3, 7	MMAT 3/5
Nelson/Nygaard Consulting Associates (2008) North America [48]	To report on the current use of wheelchairs and other mobility devices on public and private vehicles, Report	Mixed methods Review of standards and research and Qualitative (interviews, policy round table) and Quantitative (survey)	Forward-facing, 4-point tie-down, lap and shoulder belt	6, 7	MMAT 1/5
Schneider et al. (2008) North America [63]	To review the current voluntary standards for the design and performance of active containment systems, and for wheelchairs used as seats in motor vehicles, Peer-reviewed journal	Review of standards affecting containment systems in use in North America	Forward-facing, 4-point tie-down, lap and shoulder belt	5	AACODS 6/6
Brinkey et al. (2009) North America [58]	To determine knowledge about wheelchair transportation safety practices among wheelchair users and caregivers, therapists, physicians, and other professionals, Peer-reviewed journal	Quantitative-descriptive (survey)	Forward-facing, 4-point tie-down, lap and shoulder belt	7	MMAT 3/5
Karg et al. (2009) North America [18]	To review and document the status of wheelchair transportation safety, identify deficiencies and recommendations for future action, Peer-reviewed journal	Review of current practice using a workshop format	Forward-facing, 4-point tie-down and shoulder/lap belt Forward-facing, docking system Rear-facing FEB, LEB, mobility device tether	6	AACODS 6/6
Salipur and Bertocci (2010) North America [61]	To quantify active tie-down restraint loading in rear-impact testing, Peer-reviewed journal	Quantitative (simulated sled tests)	Forward-facing, 4-point tie-down, lap and shoulder belt	1	MMAT 3/5
Turkovich et al. (2011) North America [19] (same data set as van Roosmalen et al., 2011 and 2012)	To determine compliance of a prototype auto-docking system and RF-WPS to ADA maximum displacement requirements when exposed to vehicle testing, Peer-reviewed journal	Quantitative (in-vehicle testing)	Forward-facing, 4-point tie-down, lap with/without shoulder belt Forward-facing, docking system, lap with/without shoulder belt Rear-facing, FEB, automated LEB, lap belt	3	MMAT 4/5
van Roosmalen et al. (2011) North America [45] (same data set as Turkovich et al., 2011 and van Roosmalen et al., 2012)	To evaluate the usability, comfort, and independent use of two prototype wheelchair containment systems compared with the 4-point tie-down system, Peer-reviewed journal	Quantitative-descriptive (in vehicle trials and survey)	Forward-facing, 4-point tie-down, lap with/without shoulder belt Forward-facing, docking system, lap with/without shoulder belt Rear-facing, FEB, automated LEB, lap belt	7	MMAT 4/5
Ahmed et al. (2012) North America [16]	To quantify the risks posed to bus drivers while performing an active tie-down containment system procedure using traditional ergonomic analysis methods, Peer-reviewed journal	Quantitative-descriptive (ergonomic analysis of work tasks)	Forward-facing, 4-point tie-down, lap and shoulder belt	4	MMAT 2/5
Buning et al. (2012) North America [62]	To outline RESNA’s position on wheelchairs used as seats in motor vehicles, Peer-reviewed journal	Position paper summarizing research evidence and standards	Forward-facing, 4-point tie-down, lap and shoulder belt	5	AACODS 6/6
Frost et al. (2012) North America [43]	To describe the current environment, research findings, and safety-related issues associated with passengers who remain seated in their wheelchair or scooter while traveling in either fixed-route or demand-responsive LATVs, Peer-reviewed journal	Description of current systems and review of associated standards	Forward-facing, 4-point tie-down, lap and shoulder belt Rear-facing with FEB, LEB	5	AACODS 6/6
Salipur et al. (2012) North America (same data set as Frost et al., 2013) [60]	To examine video-recorded, wheelchair-related “adverse events” involving disuse and misuse of active containment systems during transit; to identify configurations associated with adverse wheelchair and passenger outcomes, Peer-reviewed journal	Quantitative-descriptive (retrospective review of video)	Forward-facing, 4-point tie-down, lap and shoulder belt	2	MMAT 5/5
van Roosmalen et al. (2012) North America [14] (same data set as Turkovich et al., 2011 and van Roosmalen et al., 2011)	To evaluate the perceived safety and usability of two prototype wheelchair containment systems compared with the 4-point tie-down system, Peer-reviewed journal	Quantitative-descriptive (in-vehicle trials and survey)	Forward-facing, 4-point tie-down, lap with/without shoulder belt Forward-facing, docking system, lap with/without shoulder belt Rear-facing FEB, automated LEB, lap belt	4	MMAT 4/5
Zaworski (2012) North America [49]	To develop and test an aisle-side containment device to enable safe and independent use of rear-facing containment, Report	Mixed Methods Review of standards and research and Qualitative (stakeholders’ needs) and Quantitative (design and development, in-vehicle testing)	Rear-facing FEB, LEB,	3, 8	MMAT 2/5
Frost et al. (2013) North America (same data set as Salipur et al., 2012) [12]	To characterize active containment system usage in public transit buses based on observations of wheeled mobility device passenger trips, Peer-reviewed journal	Quantitative-descriptive (retrospective review of video)	Forward-facing, 4-point strap tie-down, lap and shoulder belt	2	MMAT 5/5
Ahmed et al. (2014) North America [46]	To provide an understanding of factors that may pose challenges to bus operators securing a wheel mobility device passenger, Peer-reviewed journal	Quantitative-descriptive (survey)	Forward-facing, 4-point strap tie-down, lap and shoulder belt	4	MMAT 2/5
Hunter-Zaworski and Rutenberg (2014) North America [15]	To examine the use of wheeled mobility devices on LATVs, and to identify potential improvements to safety and level of service for agencies that transport larger and heavier occupied wheelchairs and scooters, Report	Review of literature, workshop, and survey of stakeholders	Forward-facing, 4-point strap tie-down, lap and shoulder belt Rear-facing, FEB, LEB, mobility device tether	6, 7	AACODS 6/6
Almada and Renner (2015) Brazil [50]	To identify ergonomics and accessibility issues faced by wheelchair users when using public transport, Peer-reviewed journal	Quantitative-descriptive (interview and survey)	Forward-facing, docking system, lap and shoulder belt	7	MMAT 4/5
Mather and Hunter-Zaworski (2016) North America [11]	To evaluate the effects of horizontal and vertical curves, speed, and driver behavior on the safety of people using wheeled mobility devices in rear-facing passive containment systems on large transit buses, Peer-reviewed journal	Quantitative-descriptive (in-vehicle testing)	Rear-facing, FEB, LEB	3	MMAT 4/5
Cross (2017) North America [42]	To review the major changes made to the ADA Accessibility Guidelines, Report	Review of guidelines	Forward-facing, 4-point tie-down, lap and shoulder belt Rear-facing, FEB, LEB	5	AACODS 4/6
Perez et al. (2019) North America [2]	To explore the usability of three commercially available containment systems in a static laboratory environment: a 4-point, forward-facing (4P-FF) containment system; a 3-point, forward-facing (3P-FF) containment system; and a semi-automated, rear-facing (SA-RF) containment system, Peer-reviewed journal	Quantitative-descriptive (in-vehicle simulations with WC users)	Forward-facing, 4-point tie-down, lap and shoulder belt Forward-facing, 3-point tie-down, lap and shoulder belt Rear-facing, FEB, automated LEB	7	MMAT 3/5
Unsworth et al. (2022) Australia [6]	To gather feedback from American (active containment systems in routine use) and Australian (few containment systems in routine use) mobility device users to guide their possible introduction in Australia	Quantitative—Descriptive survey with WC users	Forward and rear-facing, 3-point and 4-point tie-down, wheel clamps, automated systems, restraint system-lap belt with/without shoulder belts, mobility device tether	7	MMAT 4/5

* 1—Crash test; 2—Incidents; 3—Testing under normal driving conditions with different containment system scenarios; 4—Bus drivers/transit operators; 5—Review or discussion of application of standards; 6—Literature-based discussion of current practice; 7—Consumer opinion/observation; 8—Design and development. RF-WPS = Rear-Facing-Wheelchair protection System; ADA = Americans with Disabilities Act; RESNA = Rehabilitation Engineering Society of North America; FEB = Forward Excursion Barrier to limit movement to the front of the bus upon braking; LEB = Lateral Excursion Barrier to limit movement into the aisle.

**Table 2:** Summary of key findings across focus areas.

Focus Area	Author Name, Date	Key Findings
Design and development of containment systems and their use internationally	All papers included in the review, with a specific focus from the following: Hunter-Zaworski et al. (1992) [57]; Hobson and van Roosmalen (2007) [56]; Zaworski (2012) [49]	Following the introduction of the ADA in 1990, there has been wide-spread use of active containment systems such as 3-point or 4-point tie-down straps, wheel clamps, docking system, automated systems (with/without shoulder/lap belt) across North America. There is some research evidence supporting their use. Since 1995, across Europe there has been a preference for passive (or semi-active with a tether) systems where people travel in a designated containment space, rear-facing with brakes on, backrest in contact with an FEB and holding onto an aisle support in the form of an LEB, and with or without a tether. Canadian (and subsequently US researchers) have also investigated this approach since 1995, with some advocating for its use. There is very limited research evidence supporting the use of semi-active or passive containment systems.
Consumer opinion or observation of consumers using containment systems	Buning et al. (2007) [13]; Wolf et al. (2007) [59]; Nelson/Nygaard Consulting (2008) [48]; Brinkey et al. (2009) [58]; van Roosmalen et al. (2011) [45] (same dataset as Turkovich et al., 2011 and van Roosmalen et al., 2012); Almada and Renner (2015) [50]; Perez et al. (2019) [2]; Unsworth et al. (2022) [6]	In the USA and Australia, only a minority of people have ever slid or fallen while in transit, or seen a person slide or fall. In the USA, people report they have received little or no education about the proper use of containment or restraint systems, and less than 1% have knowledge that industry standards existed to guide best practice. Lack of use or the misuse of available active containment systems is very common, most probably relating to lack of time or knowledge of the bus driver. Containment systems are only rated as 7.66/10 effective in preventing injuries. Consumers prefer automated forward- or rear-facing containment systems, which are time-efficient and allow increased independence. However, they have reservations when travelling rearward with difficulties seeing approaching bus stops and concerns about motion sickness.
Testing containment systems under normal driving conditions	Hunter-Zaworski and Zaworski (2005) [47]; Hobson and van Roosmalen (2007) [56]; Wolf et al. (2007) [59]; Turkovich et al. (2011) [19] (same dataset as van Roosmalen et al., 2011 and 2012); Zaworski (2012); Mather and Hunter-Zaworski (2016) [11]	Mobility devices in normal driving situations moved less than the ADA recommendations and accelerations during hard braking and rapid turning also did not exceed ADA recommendations. Passive or semi-active containment options may be appropriate to use (noting this research is over 15 years old and technology has enabled even smoother driving with lower forces generated). Bus driver driving styles are a key consideration in how much a mobility device moves during transit.
Bus drivers’ or transit operators’ perspectives on containment systems	Spiller (1995) [41]; Schneider et al. (2008) [63]; Buning et al. (2012) [62]; Frost et al. (2012) [43]; Cross (2017) [42]; Ahmed et al. (2014) [46]	Problems noted by bus operators when using 3- and 4-point active containment systems include: o Occupational health and safety risks for bus drivers when fitting mobility devices; o Lack of training; o Difficulty identifying points for securement on a mobility device (increasing with the ever growing variety of mobility devices). Call for voluntary or mandatory standards for mobility device containment systems. Injuries to mobility device users/passengers occur when containment systems are not used. Passive or semi-active containment systems are viewed positively by bus operators.
Review of incidents involving mobility device users	Spiller (1995) [55]; Shaw and Gillispie (2003) [5]; Salipur et al. (2012) [60] (same data set as Frost et al., 2013)	LATVs are involved in relatively few crashes. Data on LATV crash incidents and outcomes are not usually publicly available. Data on adverse events for mobility device users on buses are relatively rare; however, one dataset of nearly 300 mobility device trips demonstrated incidents do occur under normal driving conditions and that misuse or no use of active containment systems was common.
Crash testing of mobility device containment systems	Hunter-Zaworski and Zaworski (2005) [47]; Hobson and van Roosmalen (2007) [56]; Salipur and Bertocci (2010) [61];	Actual acceleration rates used in testing are substantially lower than the standards recommend for crash testing, impacting the testing of containment options. Docking devices produced only minimal movement during rear-impact collisions and are recommended for use. A rear-facing containment system with an FEB but no LEB is likely to lead to a mobility device tipping over under extreme conditions.
Standards and their application	Spiller (1995) [41]; Schneider et al. (2008) [63]; Buning et al. (2012) [62]; Frost et al. (2012); Cross (2017) [42]	Voluntary practice standards to guide the design and testing of containment systems exist in the USA. Researchers are calling for: o Standards for containment systems that reflect current modern low-floor LATVs and modifications to the ADA to take into account different vehicle environments; o Best practice guidelines/or training tools to educate (i) passengers on how to use a mobility device on public transport and (ii) bus drivers concerning correct use of containment systems; o A certification process that identifies transit operators with appropriate training for staff; o Random monitoring of transit operators regarding appropriate use of containment systems.

ADA = Americans with Disabilities Act; FEB = Forward Excursion Barrier to limit movement to the front of the bus upon braking; LEB = Lateral Excursion Barrier to limit movement into the aisle; LATVs = Large Accessible Transit Vehicles.

## 4. Discussion

There is a relatively small literature on containment systems for mobility devices on LATVs, with only 33 papers across the white and grey literatures meeting the criteria for inclusion in this review. Most documents included (n = 30) have been generated by North American researchers, with the introduction of the ADA prompting researchers to evaluate the use of three-point and four-point tie-down systems and to also report on the development and testing of new prototypes for forward- and rearward-facing containment. Different research groups across the US have worked with local transit authorities to adopt differing approaches to containment for people using mobility devices, resulting in competitive innovation but limited consensus on the best approach to use that complies with the relevant standards. It is also possible that some US researchers have had a financial investment in developing containment prototypes, thus compromising independent investigation. In contrast, there is almost no literature from European researchers, published in English, on the topic of mobility device containment on LATVs, particularly with regard to passive or semi-active options. It appears that bus regulators and operators settled on rearward-facing containment systems with FEBs, LEBs, and the option of tethers and that there have never been sufficient complaints or incidents to instigate a review of these practices by research teams.

As it has been over 15 years since the workshop was convened in the US on mobility device containment research [18], it is time for another event to bring together key stakeholders including researchers, commercial enterprises designing and installing containment systems, transport operators, and consumers to discuss requirements to propel the field forward. While meetings such as this can identify and promote a consistent and strategic research agenda, the recommendations provided below are also made. Furthermore, such meetings should be co-designed and co-facilitated by consumers with lived experience of mobility device use on public transport. Further research examining the perspectives and experiences of consumers on all aspects of the design, adoption, and use of containment systems should also be co-designed and conducted.

A review of international guidelines and standards is required to identify what is currently recommended or mandated internationally, and how standards might be supporting or hindering practice. While some documents included in this review cite the relevant International Standards Organisation (ISO) or US-specific standards underpinning their work [2,19], a dedicated systematic review of international documents including Europe, the US, Canada, Brazil, and Asia Pacific countries such as Japan and Australia is required. Comparing and contrasting guidelines and standards from the perspective of different stakeholders and including considerations of the practical advantages and disadvantages of applying these, the conflicts that exist between crashworthiness requirements and government regulations, and whether the standards are voluntary or enforced is valuable to identify where gaps or problems exist and what research is required to lead to revisions in these documents.

Technological advancements in bus design and improved road conditions have potentially led to smoother transportation with fewer crashes or sliding or tipping incidents for people using mobility devices. However, there is relatively little documentation of incidents or crashes due to the commercially sensitive nature of such data. A deidentified national database could be established by transport authorities to gather evidence to support the need for further investigations into containment systems. Data documenting the g forces generated during everyday driving as well as during specific maneuvers, such as harsh braking and cornering on inclined/declined slopes, is also required, given that buses are more stable than ever and drivers often have advanced instrumentation to alert them to maneuvers likely to cause difficulties for ambulant passengers as well those using mobility devices. The g forces listed in current guidelines and standards are likely out of date, and certainly lack translation from crash testing data using WC19 to outcomes for people using modern ultra-light-weight manual wheelchairs as well as high-tech power wheelchairs.

Further research into the design of containment systems has been repeatedly called for [2,11,18], but crash testing of such systems, as well as determining their compliance with design rules, standards, and guidelines, coupled with fear of litigation following adverse incidents, has certainly hampered innovation in this area. Innovation may also have been hampered by the need to commercialize containment systems, producing competing interests between researchers and manufactures. New automated designs have been introduced, but independent documentation of their effectiveness is limited [2], their cost may be prohibitive for many bus operators, and users may not be able to meet use requirements. For example, the most frequently installed semi-automated system in the US requires the user to position themselves precisely to allow the system to operate. Independent research validating the use of such systems is yet to be published. While the effectiveness of three- and four-point tie-down systems when used correctly has been well-established, further research is also required to continue to test the effectiveness of passive and semi-active systems using FEBs, LEBs, and the possible addition of a tether belt for the mobility device. The independence afforded to users by these systems [11], as well as their ability to accommodate the ever-growing number of designs of mobility devices, demands they receive further consideration.

Finally, although this review was rigorous in its approach to identifying and appraising the literature, several limitations exist. The reviewers were only able to access and appraise literature presented in English, and additional grey literature may exist that was not indexed in the databases searched, although our hand searching of reference lists was important to minimize this limitation. This review was conducted by two authors from outside North America. While this may have limited the presentation of the historical development of active containment systems and input to the quality appraisal, this approach was advantageous to limit bias in terms of both reporting on and appraising the literature using the AACCODS and MMAT scoring, since neither author is affiliated with any of the research groups working in this area.

## 5. Conclusions

The need for people using mobility devices to travel on public buses is not only based on convenience and economic necessity but is also enshrined in legislation in most developed countries. While access to board and position within an LATV is the first requirement of the journey, ensuring the journey is also safe for people using mobility devices poses many challenges given that such passengers are not travelling on seats bolted to the floor, like ambulant passengers are. A range of active, semi-active, and passive containment systems exist, yet each poses challenges and advantages relating to the degree of protection offered during emergency maneuvers or in a crash, the mobility device user’s skill requirements, user dignity and independence, bus driver involvement and injury risk in fitting active systems, impact on dwelling times, and costs associated with installation and maintenance. The findings from this systematic review of literature in the field of mobility device securement on LATVs reveal that although the quality of documents is good, there is limited contemporary research in this field, particularly relating to the use of passive and semi-active containment systems. Further investigations are also required to document the forces that impact mobility devices when travelling in modern high-tech, low-floor buses to determine optimal FEB and LEB design and positioning. Transport planners seek the most efficient ways for people to enjoy the freedoms associated with low-cost public transport, but with increasing numbers of people using mobility devices, and particularly more older people using mobility scooters, the challenge is to identify the best securement options for people using these devices to promote safe travel for all. Contemporary policies and guidelines can then be developed to support people using mobility devices to have a range of options to promote safe travel on public buses.

## Data Availability

Not applicable.

## Appendix A. MEDLINE Search Strategy

1. NOFT (“public transport*” OR “public transit bus*” OR “large access* transit vehicle*” OR LATV*) limited by year 1990–2020 (4379 results)
2. MH “Motor Vehicles” limited by year 1990–2020 (5643 results)
3. 1 OR 2 (9843 results)
4. NOFT (“Wheelchair tie down*” OR “Wheelchair tiedown*” OR “Wheelchair tie-down*” OR “wheelchair occupant restraint system*” OR wheelchair* OR “wheelchair safety” OR “occupant restraint*” OR “wheelchair transport* safety” OR WTORS OR “wheelchair secure*” OR “passenger safety” OR “mobility aid restraint system*” OR wheelchair secure* system*) limited by year 1990–2020 (8207 results)
5. MH “Wheelchairs” limited by year 1990–2020 (4193 results)
6. 4 OR 5 (7230 results)
7. 3 AND 6 (125 results)

## Appendix B. Grey Literature Search Strategy

Data bases searched:

1. Google Search (first 50)
2. Government

Japan, www.japan.go.jp (accessed on 30 May 2022)

United Kingdom, www.gov.uk/government/organisations/department-for-transport (accessed on 30 May 2022)

America, www.transportation.gov/ (accessed on 30 May 2022)

Canada, www.tc.gc.ca/en/transport-canada.html (accessed on 30 May 2022)

Australia, www.infrastructure.gov.au/transport/ (accessed on 30 May 2022)

Sweden, www.government.se (accessed on 30 May 2022)

3. Transport databases

National Transportation Library, ntl.bts.gov/ntl/ (accessed on 30 May 2022)

Transport Research Information Documentation, trid.trb.org/ (accessed on 30 May 2022) (first 25)

Transport Research Board, www.trb.org (accessed on 30 May 2022)

Society of Automotive Engineers, www.sae.org (accessed on 30 May 2022) (first 20)

National Council on Disability, www.ncd.gov/ (accessed on 30 May 2022) (first 20)

Rehabilitation Engineering Society of North America www.resna.org/ (accessed on 30 May 2022) (first 15)

4. Grey literature databases

Open Grey-grey literature in Europe www.opengrey.eu (accessed on 30 May 2022)

Grey Guide-repository of grey literature greyguide.isti.cnr.it/ (accessed on 30 May 2022)

National Repository of Grey Literature www.nusl.cz/?lang=en (accessed on 30 May 2022)

5. Transport Research databases

Australasian Transport Research Forum www.australasiantransportresearchforum.org.au/ (accessed on 30 May 2022)

European Transport Research Alliance www.etralliance.eu/ (accessed on 30 May 2022)

Asian Transportation Research Society www.atransociety.com/atrans-website/home.html (accessed on 30 May 2022)

Nordic Road and Transport Research www.nordicroads.com/ (accessed on 30 May 2022)

## Appendix C. Examples of Containment Systems as Described in Reviewed Documents Including (as Provided in Table 1, Column 4): Forward- or Rear-Facing, Rearward Padded Backboard as a Forward Excursion Barrier (FEB), Containment System-Strap Tie-Downs, Wheel Clamps, Aisle Barrier in the Form of a Lateral Excursion Barrier (LEB), Docking System, Restraint System Lap Belt with/without Shoulder Belts, Tether Belt for Wheelchair, and Automated Systems

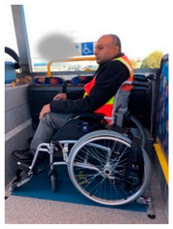
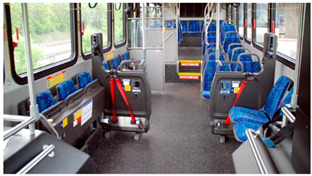
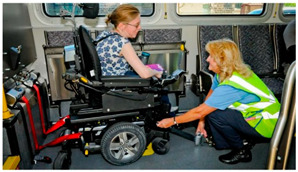

		
Forward-facing, 4-point tie-down with seatbelt. Source: authors.	Forward-facing, 3-point tie-down with seat belt. Source: https://www.metrotransit.org/new-buses-bring-new-look-both-inside-and-out (accessed on 1 June 2021)	Forward- or rearward-facing, 4-point tie-down with additional bus seatbelt (lap sash securing person, to wheelchair to bus) Source: https://www.valleymetro.org/bus-accessibility (accessed on 1 June 2021)

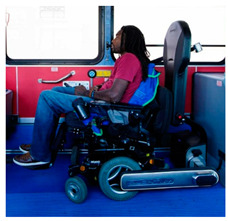
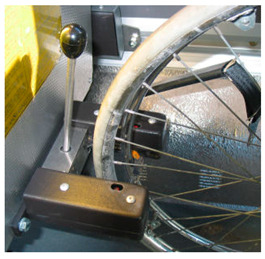
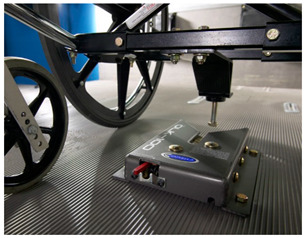
		
Rearward-facing, with FEB, automated securement and seatbelt. Source: https://www.qstraint.com/en-gb/quantum/ (accessed on 1 June 2021)	Wheel clamp. Source: https://winnipegtransit.com/en/rider-guide/accessible-transit/ (accessed on 1 June 2021)	Forward-facing, docking system. Source: https://www.ridc.org.uk/features-reviews/out-and-about/wheelchair-accessible-vehicles-wavs-0/wheelchairs-wavs (accessed on 1 June 2021)
****		
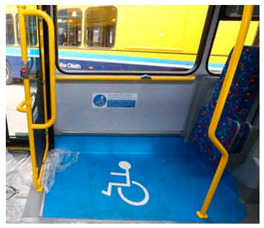
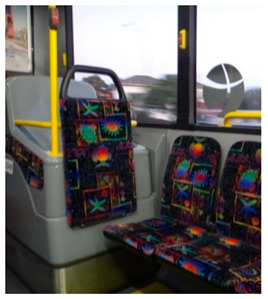
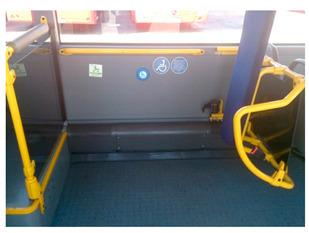
Rearward-facing FEB with aisle stanchion as LEB. Source: https: witter.com/IrishWheelchair/status/1085144789861969922 (accessed on 1 June 2021)	Rearward-facing, FEB. Source: http://www.sgbuses.com/picture.php?/39852-b7rle_optimus_int_sideseats/category/transperth-b7rle-optimus (accessed on 1 June 2021)	Rearward-facing FEB, with LEB and seatbelt. Source: https://evobusspainblog.wordpress.com/2016/07/ (accessed on 1 June 2021)
****		****
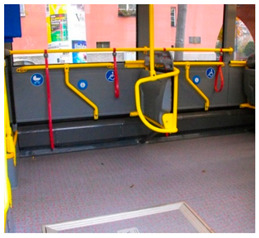
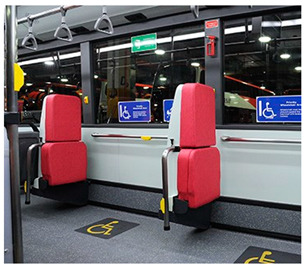
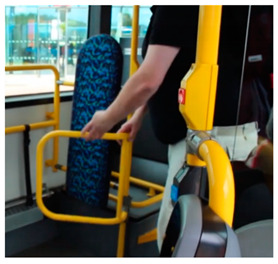
Rearward-facing FEB, with LEB and hanging hand-held tethers. http://bmin.info/WP/2017/10/ (accessed on 1 June 2021)	Rearward-facing FEBs, with fold-up LEBs. Source: https://www.lta.gov.sg/content/ltagov/en.html (accessed on 1 June 2021)	Rearward facing FEB, with foldout LEB and hand-held tether. Source: https://www.youtube.com/watch?v=6lbQjfGIhd4. (accessed on 1 June 2021)
